# Epigenetic therapy targeting bone marrow mesenchymal stem cells for age-related bone diseases

**DOI:** 10.1186/s13287-022-02852-w

**Published:** 2022-05-16

**Authors:** Yi Zhao, Jiawei He, Tao Qiu, Haoyu Zhang, Li Liao, Xiaoxia Su

**Affiliations:** State Key Laboratory of Oral Diseases & National Clinical Research Center for Oral Diseases & Department of Pediatrics & National Engineering Laboratory for Oral Regenerative Medicine, West China Hospital of Stomatology, Sichuan University, Chengdu, China

**Keywords:** Epigenetics, Aging, Mesenchymal stem cell (MSC), Therapy, Age-related bone diseases

## Abstract

As global aging accelerates, the prevention and treatment of age-related bone diseases are becoming a critical issue. In the process of senescence, bone marrow mesenchymal stem cells (BMSCs) gradually lose the capability of self-renewal and functional differentiation, resulting in impairment of bone tissue regeneration and disorder of bone tissue homeostasis. Alteration in epigenetic modification is an essential factor of BMSC dysfunction during aging. Its transferability and reversibility provide the possibility to combat BMSC aging by reversing age-related modifications. Emerging evidence demonstrates that epigenetic therapy based on aberrant epigenetic modifications could alleviate the senescence and dysfunction of stem cells. This review summarizes potential therapeutic targets for BMSC aging, introduces some potential approaches to alleviating BMSC aging, and analyzes its prospect in the clinical application of age-related bone diseases.

## Background

As global life expectancy increases, the population framework ages rapidly, leading to increased medical costs [1]. 23% of the total burden of global diseases comes from age-related diseases of 60-year-olds and over of the population [2], rendering aging a major public health problem. Aging is a complex syndrome, accompanied by a variety of age-related diseases and an increased risk of adverse events [3, 4] in the cardiovascular, skeletal and muscular, nervous, and endocrine systems [5–8]. Among diseases of the 60-over population, skeletal and muscular diseases account for 7.5%, which is one of the main age-related diseases [2]. These diseases result in disability rather than death, causing long-term care costs and excessive health expenditure. Up to now, various treatments have been studied to prevent and treat age-related bone diseases. In this review, epigenetic therapy targeting BMSCs for age-related bone diseases is summarized. We firstly searched PubMed and Web of Science with keywords “mesenchymal stem cells or mesenchymal stromal cells” and “osteoporosis or aging” and “epigenetics, histone, DNA methylation, microRNA, or non-coding RNA.” We then selected the relevant documents on the topic after reading the abstract and performed a systemic review of these documents.

## Main text

### Exhaustion and dysfunction of bone marrow MSCs during aging

The term mesenchymal stem cell (MSC) was introduced by Caplan in 1991. It is a kind of stem cell that originated from the embryonic mesoderm and can be isolated from many tissues, such as bone marrow, fat, umbilical cord, uterus, peripheral blood, dermis, muscle, synovium, tonsil, periodontal ligament, and dental pulp [9]. In general, MSCs possess the capacities of self-renewal and multipotent differentiation, which are essential capacities of adult stem cells. As one of the most prominent MSCs, bone marrow mesenchymal stem cells (BMSCs) have potent self-renewal capability and can differentiate into multiple cell types, including osteoblasts, chondrocytes, and adipocytes [10]. By differentiating into progenitors of osteoblasts and chondrocytes, BMSCs are critical in bone development and remodeling. BMSCs also contribute to bone tissue homeostasis through immune regulation and anti-inflammatory effects, and in some cases, with antioxidant and angiogenesis activity [11, 12].

In the process of aging, various disorders and dysfunctions appear in BMSCs. During senescence, the proliferation capability of BMSCs decreased both in vivo and in vitro. BMSCs from the younger donors proliferate faster than those from the older groups [13]. Long-term passage also decreased the proliferation ability of BMSCs in vitro [14]. Moreover, aging BMSCs tend to differentiate into adipocytes rather than osteoblasts, which reduces bone self-repairment capability and bone mineral density [15]. Compared with younger people, the immunomodulatory and anti-oxidative ability of elderly individuals' BMSCs also decreases, which may lead to accumulation of toxic metabolites and increase the risk of bone homeostasis disorder [16, 17]. In a recent experiment, BMSCs were isolated from the subchondral bone of three groups of people (osteoarthritis patients, osteoporosis patients, and healthy donors). Analyzing and comparing the isolated MSCs, it is found that BMSCs of patients with the two diseases have defects in osteogenesis and chondrogenesis. And also in this study, the expression of leptin receptor is low in the bone-derived BMSCs of patients with osteoarthritis. Since leptin receptor is a marker of stem cells in the adult bone marrow, the finding indicates the exhaustion of skeletal muscle and bone-derived BMSC is a sign of osteoarthritis. Therefore, the treatment of exhausted BMSCs in patients with osteoarthritis (OA) and osteoporosis (OP) is important [18].

On account of the crucial role of BMSCs in bone function, BMSCs have attracted increasing attention in bone disease treatment. Treatment such as transplantation of BMSCs [19–22], BMSC-derived exosomes therapy [23], and drugs regulating the function of BMSCs showed positive therapeutic effects in preclinical studies, suggesting that BMSCs are potential targets of age-related bone disease treatment.

### Epigenetic alteration and aberration during BMSC aging

Epigenetic modifications exert a profound influence on the fate of BMSCs at multiple levels by regulating gene expression. These modifications are reversible but can be stably passed down along cell lineages [24]. Epigenetic modifications can be added by specialized enzymatic “writers,” removed by “erasers” and recognized by “readers.” Generally, the modifications include DNA methylation, histone modification, chromatin remodeling, and posttranscriptional processing [25]. Posttranscriptional processing mainly refers to RNA modification, such as message RNA (mRNA) modification, micro-RNA (miRNA) and long noncoding RNA (lncRNA) expressing regulation.

During the aging process of BMSCs, epigenetic modification gradually changes in response to exogenous and endogenous factors [25, 26]. Hormonal, immunologic, and metabolic factors are the critical microenvironmental signals contributing to the dysfunction of BMSCs during aging. As estrogen deficiency occurs, transcription of BMSCs was influenced by altered histone modification. In the inflammatory microenvironment, proinflammatory cytokines synergistically induce differentiation disorders of BMSCs. The underlying mechanisms involve posttranscriptional regulations by micro-RNAs and cross talk of key signaling. In hyperglycemia, sonic hedgehog signaling and reactive oxygen species are involved. Finally, these changes interact together to influence the transcription factors Runx2 and peroxisome proliferator-activated receptor gamma, which, respectively, regulate osteogenesis and adipogenesis of BMSCs [27].

During BMSC senescence, DNA methylations are reduced in the whole genome [28]. Meanwhile, inhibitory histone modifications such as H3 Lys9 trimethylation and H4 Lys20 trimethylation are also reduced [29]. The reduced inhibitory modifications are centered on the region of the heterochromatin, which may lead to genome instability. In addition, the lack or overexpression of some RNAs that are closely related to RNA-regulated enzymes will accelerate aging. The gradual accumulation of age-related epigenetic changes would destroy the normal state of gene expression and numerous signaling pathway cascades, leading to gradual loss of cellular homeostasis and dysfunction of BMSCs [24, 30].

### Potential epigenetic target for BMSC aging therapy

Since epigenetic modifications are reversible, epigenetic changes during BMSC aging provide potential targets for treatment. Due to the capacity of self-renew, MSCs exist for the long term in bone tissue, which facilitates the accumulation of aberrant epigenetic modifications in them. Although osteoblasts and osteoclasts are critical regulators of bone homeostasis, they are terminal cells that could only survive for a short term and could not inherit the aberrant epigenetic modifications to the descendant cells. Furthermore, the exhaustion and dysfunction of stem cells have been demonstrated as one of the essential hallmarks of aging. Therefore, the majority of studies focus on epigenetic modifications on aged MSCs and have shown promising therapeutic effects for age-related bone diseases (Table 1).

**Table 1:** Epigenetic targets of MSC aging

Treating target	Promotion therapy site				Inhibition therapy site			
DNA Methylation	TET1/2 ALKBH1 DNMT3B	[31] [32] [33]			DNMT1 DNMT3A DNMT3B	[34] [34, 35] [36]		
Histone Modification	G9a KDM4A KDM4B GCN5	[37] [38] [39] [40]			SUV39h1/2 EZH2 KAT7 KDM5A	[41] [42–44] [45] [46]	LSD1 Jarid1a HDAC1/2	[47] [48] [49, 50]
Chromatin Remodeling	CBX4 ZKSCAN3	[51] [52]			BRM	[53]		
mRNA Modification	METTL3 FTO	[54–56] [57–59]						
miRNA Expression	miR-21(-5p) miR-26a/b miR-27b miR-29a-3p miR-30c-5p miR-34a miR-130a miR-148 miR-328-3p miR-335-5p	[60, 61] [62] [63] [35] [35] [64, 65] [63, 66] [67] [68] [69]	miR-5106 miR-9 miR-17-5p miR-27a miR-199b-5p miR-217 miR-218 miR-346 miR-433-3p miR-590-3p	[70] [71, 72]	miR-31(a-5p) miR-34a miR-132-3p miR-140 miR-188 miR-214 miR-222 miR-7a-5p miR-9-5p miR-16–2-3p miR-23a/b miR-24 miR-27a miR-30 miR-93-5p miR-98 miR-100 miR-125b miR-135a	[73, 74] [64] [75] [76] [77] [76] [78] [71, 72]	miR-137(-3p) miR-139-5p miR-143 miR-153 miR-154-5p miR-203 miR-203-3p miR-204 miR-205 miR-217 miR-218 miR-221 miR-338 miR-338-3p miR-378 miR-381 miR-708 miR-1297	[71, 72]
lncRNA	LINC00341 lncRNA- ENST0000563492	[79] [80]			lncRNA- MIR31HG	[81]		
					lncRNA CIR	[82]		

Epigenetic targets of MSC aging include DNA methylation, histone modification, chromatin remodeling, and RNA modification. Potential therapy sites that need to be promoted or suppressed are listed above

TET, ten-eleven translocation family protein; ALKBH, AlkB homolog; DNMT, DNA methyltransferase; G9a, a lysine-specific histone methyltransferase; KDM, lysine-specific demethylase; GCN, also known as Eif2ak4, eukaryotic translation initiation factor 2 alpha kinase 4; SUV39H, a lysine-specific histone methyltransferase; EZH, enhancer of zeste homolog; KAT7, a histone acetyltransferase; LSD, also known as KDM, lysine-specific demethylase; Jarid1a, Jumonji AT-rich interactive domain 1a; HDAC, histone deacetylase; CBX4, chromobox 4; ZKSCAN3, zinc finger with KRAB and SCAN domains 3; BRM, brahma, the subset of SWI/SNF (a chromatin remodeling complex); METTL, methyltransferase-like; FTO, fat mass and obesity-associated protein

### Treating target for DNA methylation

DNA methylation mostly occurs on the fifth carbon of cytosine in CpG islands. These islands are mainly concentrated in the promoter region of genes and are usually unmethylated. When they are methylated, the interaction between DNA and transcription factors is prevented, leading to gene silence. In physiological conditions, genomic DNA methylation remains stable in the long term. But significant differences were observed at specific CpG sites during aging [83]. Methylations in most CpGs are reduced, while are increased in only a minority of CpGs in aged stem cells. The region with reduced methylation is predominantly heterochromatin, leading to loose chromosomes and genomic instability during aging. The minor sites with increased methylation are mainly CpG-rich regions in the promoter of genes related to growth control and tumor inhibition, leading to inhibition of gene expression and deterioration of cell function. This causes gradual impairment of homeostasis and increases the risk of age-related diseases.

DNA methylation is catalyzed and maintained by the DNA methyltransferase (DNMT) family. DNMTs in mammals are divided into two families: DNMT1 and DNMT3 (DNMT2, mainly methyltransferase of tRNA) [28]. DNMT3 includes two methyltransferases DNMT3a, DNMT3b, and a regulatory protein DNMT3L. Experiments have shown that inhibition of DNMT1, DNMT3A, DNMT3B in BMSCs can resist aging, promote angiogenesis, and suppress cancer. After inhibiting DNMT3A, the methylation of CpG islands was reduced in upstream of Sod2 gene, leading to the expression of superoxide dismutase 2 (SOD2), a dominant antioxidant enzyme for anti-aging [35]. Knockdown of DNMT1/DNMT3a in BMSCs induced the expression of several arterial-specific transcription factors with high angiogenesis activity [34]. Inhibition of DNMT3B can reduce DNA methylation in the promoter of tumor suppressor genes PTEN [36]. However, contrary to DNMT1, DNMT3A, and DNMT3B, inhibition of DNMT3l leads to hypermethylation and inhibition of some genes related to homeostasis, which reduce osteogenesis of BMSCs in vitro [84]. Therefore, DNMT inhibitors specific to DNMT1, DNMT3A, and DNMT3B may provide a potential therapeutic effect on BMSC dysfunction and bone aging. Conversely, a study has shown that DNMT3b, not DNMT3a, decreases in OA mouse models and chondrocytes of OA patients, and gaining function of DNMT3b in murine articular chondrocytes showed a chondroprotective effect [33]. These contradictory results suggest further researches are needed to confirm the mechanisms of DNMT in aging regulation.

Ten-eleven translocation (TET) protein catalyzes DNA demethylases, which converts s5-methylcytosine (5-mC) to 5-hydroxymethylcytosine (5-hmC) as an intermediate step for removing methylation markers [25]. TET protein family includes TET1, TET2, and TET3 [28]. TET1 is an indirect regulator of osteogenesis and adipogenesis of BMSC, which probably works via recruiting other epigenetic modifiers such as SIN3A and EZH2. On the contrary, TET2 directly regulates BMSC osteogenesis and adipogenesis. The effect of TET3 was not obvious in BMSCs. Researches have shown that TET1 and TET2 decrease gradually at the same time in OP. In the experiment, the knockout of mouse *Tet1* and *Tet2* genes decreased the levels of 5hmC and impaired bone formation ability of BMSCs [31], indicating that increasing TET in BMSC may provide new therapeutic strategies to prevent bone loss and promote the recovery of age-related bone diseases. However, opposite experimental results exist. Lack of TET2 in BMSCs can increase proliferation and renewal ability and enhance the osteogenic differentiation potential of BMSCs, leading to the progression of bone marrow malignancies [85]. Therefore, the conclusion remains to be drawn by further studies.

AlkB protein is part of the adaptive response mechanism to repair DNA alkylation damage. It participates in the repair of DNA damage through the oxidative demethylation of 1-methyladenine and 3-methylcytosine. AlkB homolog 1 (ALKBH1) catalyzes the demethylation of DNA N6-methyladenine (N6-mA). The increased expression of ALKBH1 in BMSCs can reduce the levels of genomic DNA N6-mA, increase the expression of osteogenesis-related genes, enhance the activity of alkaline phosphatase, and promote bone mineralization [32]. ALKBH1 is indispensable for BMSC osteogenic differentiation, suggesting that it is another potential therapeutic target.

## Treating target for histone modification

Histone modification refers to the post-translational modification of histones. Most histone modifications are involved in the first 30 amino acid sites of the N-terminal domain of histones, such as H3K4, H3K9, and H3K27 [25]. Histone modification types are diverse, including methylation, acetylation, phosphorylation, ubiquitination, SUMOylation, and proline isomerization, of which methylation and acetylation are the main types [29]. Histone methylation is mediated by histone methyltransferase and histone demethylase, and histone acetylation is modified by histone acetyltransferase and histone deacetylase (HDAC).

Histone modification has either inhibitory or active effects. Inhibitory modification compresses heterochromatin and inhibits gene expression (e.g., H3K9 and H3K27 methylation). Active modifications enable the DNA chain to unwind more easily and promote gene expression (e.g., H3K4 methylation, H3K9, and H3K14 acetylation). This usually corresponds with the trend of DNA methylation mentioned above for heterochromatin modification. For example, high levels of DNA methylation are associated with inhibitory modification of H3K9me3 and H3K27me3, while low levels of DNA methylation are associated with active modifications of H3K4me1. It is generally believed that, during BMSC senescence, inhibitory histone modification H3K9me3 and H3K27me3 decrease, resulting in instability of chromatin structure, disorders of BMSC gene expression, and disturbed homeostasis [25, 88, 89].

Histone modifications on different sites are mediated by different enzymes; thus, regulating specific histone modification enzymes can modulate BMSC nucleus homeostasis and combat BMSC aging. For example, EZH2 catalyzes the inhibitory modification H3K27me3. Suppression of EZH2 prevents BMSCs from differentiating into adipocytes rather than osteoblasts, resulting in increased bone formation during OP [42]. Knockdown of SUV39H1, a methyltransferase of H3K9me3, or its auxiliary factor HP1α will down-regulate H3K9me3 and lead to BMSC senescence, while overexpression of HP1α would up-regulate H3K9me3 level, thus promoting BMSC osteogenic differentiation [41]. The regulation modes of histone modification enzymes (summarized in Table 2) can provide viewpoints for further exploration.

**Table 2:** Histone modification and enzyme mechanism

Enzyme	Histone modification		Heterochromatin	Gene expression	Promoting osteogenesis	References
	Sites	Types				
G9a SUV39h1/2	H3K9	Methylation	Stable	Down	↑ ↓	[37] [41]
KDM4B	H3K9	Demethylation	Unstable	Up	↑	[39]
EZH2	H3K27	Methylation	Stable	Down	↓	[42]
KDM6B	H3K27	Demethylation	Unstable	Up	–	
/	H3K4	Methylation	Unstable	Up	–	
KDM5A Jarid1a LSD1	H3K4	Demethylation	Stable	Down	↓ ↓ ↓	[46] [48] [47]
GCN5	H3K9	Acetylation	–	Up	↑	[40]
KAT7	H3K14	Acetylation	–	Up	–	[45]
HDAC1/2	H2A H2B H3 H4	Deacetylation	Stable	Down	↓	[50, 86, 87]

Histone modification enzymes mediate histone methylation, acetylation, and other modification types in different sites. Active modifications can enable the DNA chain to unwind more easily and promote gene expression, while inhibitory modification can compress heterochromatin and inhibit gene expression. Active modification of age-related genes will accelerate BMSC aging and suppress osteogenesis, and active modification of anti-aging genes is carried out to promote osteogenesis and decelerate senescence; inhibitory modification, vice versa

G9a, a lysine-specific histone methyltransferase; SUV39H, a lysine-specific histone methyltransferase; KDM, lysine-specific demethylase; EZH, enhancer of zeste homolog; Jarid1a, Jumonji AT-rich interactive domain 1a; LSD, also known as KDM, lysine-specific demethylase; GCN, also known as Eif2ak4, eukaryotic translation initiation factor 2 alpha kinase 4; KAT7, a histone acetyltransferase; HDAC, histone deacetylase

However, aging remains a complex process, and epigenetic regulatory effects differ according to differently modified genes. To some extent, histone modification is similar to a “switch” of genes, denoting that active modification of age-related genes will accelerate BMSC aging, and active modifications of anti-aging genes play an anti-aging role. Both G9a and SUV39h1/2 could catalyze the methylation of H3K9, but the same inhibitory histone modification H3K9me3 produces different results. G9a inhibitors (e.g., A366, BIX01294) promote adipogenesis [37], but SUV39h1/2 inhibitors (e.g., chaetocin) promote osteogenesis [41]. In conclusion, different inhibitors exert different effects, which is critical and calls for researchers’ attention in selecting inhibitors.

Another example is HDAC inhibitors. The HDAC family contains 11 HDACs, which are divided into four enzyme classes I, IIa, IIb, and IV. Histone deacetylase HDAC1 and HDAC2 take effect indirectly via regulating the increase in histone methylase and the decrease in histone demethylase. Studies showed that trimethylated H3K9 and H3K27 lead to stable heterochromatin and down-expressing of genes. It should be mentioned that HDAC1 and HDAC2 decrease as BMSC ages, while HDAC4, HDAC5, and HDAC6 increase during BMSC senescence. The indirect effects and contradictory changes may result in the current conflict between HDAC inhibitors for therapy of BMSC aging. For example, HDAC inhibitors trichostatin A (TSA) and largazole, can decelerate BMSC senescence; TSA, vorinostat, and entinostat/MS-275 can promote osteogenesis of BMSCs [49, 50, 86, 87], suggesting application possibility for BMSC aging therapy. But other HDAC inhibitors, including sodium butyrate (NaBu), VPA, and entinostat/MS-275, can accelerate BMSC senescence, demanding further research to study the side effects of HDAC inhibitors [29].

### Treating target for chromatin remodeling

Before genes are transcribed, chromatin is transformed into a looser state. This process requires the participation of the chromatin remodeling complex, which uses ATP as energy to change the position and composition of nucleosomes, thereby regulating chromatin structure and gene expression [90, 91]. SWI/SNF is a type of chromatin remodeling complex, while mammalian brahma (BRM), an ATPase, is a component of SWI/SNF. Recent studies have shown that the knockdown of *Brm* gene promotes BMSC osteogenesis and may be a potential therapeutic target [53]. In addition, CBX4 protein is down-regulated during aging, resulting in unstable nucleolar heterochromatin, increased ribosome production and protein translation, and accelerated cell senescence. The overexpression of CBX4 delayed the senescence of hMSCs and reduced the development of OA in mice [51]. What’s more, zinc finger protein with KRAB and SCAN domain 3 (ZKSCAN3) has been considered as the main inhibitor of autophagy for a long time. Recent studies have determined the new role of ZKSCAN3 in reducing aging, which is independent of its autophagy-related activity. ZKSCAN3 maintains heterochromatin stability by interacting with heterochromatin proteins and laminin. Down-regulation of ZKSCAN3 was observed in aging hMSCs, and the depletion of ZKSCAN3 accelerated the senescence of these stem cells [52].

### Treating target for RNA modification

#### mRNA modification

Modification of m6A on mRNA is relative to “writer” (including methyltransferase-like3 (METTL3), methyltransferase-like14 (METTL14), and Wilm’s tumor-associated protein (WTAP)), “eraser” (involving demethylases FTO and ALKBH5), and “reader” (the effector proteins that can recognize m6A) [92, 93]. The loss of METTL3 function leads to insufficient osteogenic differentiation, increased bone marrow fat, and bone formation defects, indicating that METTL3 is important for BMSC osteogenesis [54–56]. FTO is significant for normal bone growth and mineralization. Researches have shown that inhibition of FTO expression, such as IOX3 treatment, can reduce bone mineral density, change the distribution of adipose tissue, and increase the risk of fracture, which may be an important therapeutic target for BMSC aging of age-related bone diseases [57–59].

#### miRNA expression

Micro-RNAs (miRNAs) are small, single-stranded noncoding RNAs (ncRNAs) of usually 21–25 nucleotides (nt) long. They account for 1–5% of the human genome and regulate 30–60% of protein-coding genes. miRNAs play a crucial role in gene regulation via binding to the 3′-untranslated region (3′-UTR) of target mRNA, which will be degraded or silenced subsequently. miRNAs can regulate the proliferation, differentiation, and apoptosis of BMSCs [71]. The regulatory targets of miRNA for BMSCs are mainly genes in the pathways for promoting or inhibiting osteogenesis, respectively, which lead to positive or negative regulatory effects. For example, miRNA-21 promotes BMSC osteogenesis [60], while anti-miR-31 improves BMSC osteogenic differentiation [73]. Other potentially valuable miRNAs in epigenetic therapy are listed in Table 1. The expression of miRNA could be regulated by directly transporting miRNA mimics or inhibitors in vitro and in vivo [72]. To efficiently regulate the levels of miRNAs for anti-aging or osteogenesis, miRNA mimics or inhibitors can be delivered via various carriers, such as nanoparticle and extracellular vesicles.

#### Long noncoding RNA

Long noncoding RNA (lncRNA) is a type of noncoding RNA with a length of more than 200 nucleotides. LncRNA lacks the open reading frame to encode proteins and has almost no protein-coding function [94]. But they can regulate gene expression and may be involved in various critical functions and activities of BMSCs, including osteogenesis and cellular senescence. Up-regulation of certain lncRNAs can promote bone formation. For example, studies have shown that overexpression of LINC00341 enhances the expression of osteogenic genes [79]. lncRNA ENST00000563492 promotes osteogenic differentiation of BMSCs by up-regulating CDH11. LncRNA ENST0000563492 can also improve the osteogenesis–angiogenesis coupling process by enhancing VEGF expression [80]. On the contrary, knockdown of some lncRNAs can promote bone formation. For example, MIR31HG interacts with NF-κB to regulate bone formation and inflammation. Knockdown of MIR31HG not only promotes osteogenic differentiation but also counteracts inflammation-induced inhibition of osteogenesis of MSCs derived from adipose tissue [81]. LncRNA is not only influential in bone formation, but also in cartilage formation. Basic helix–loop–helix (bHLH) transcription factors may regulate gene expression in the cell lineage during the embryo and after birth. Atonal homolog 8 (ATOH8), a member of the bHLH transcription factor family, is involved in embryogenesis and the development of various tissues. In the process of cartilage differentiation of hUC-MSC, lncRNA CIR is down-regulated, while ATOH8 is up-regulated. The combination of lncRNA CIR and EZH2 promotes the methylation of ATOH8 and inhibits the expression of ATOH8. Therefore, knocking down lncRNA CIR or overexpressing ATOH8 can promote BMSC differentiation and chondrogenesis [82]. These findings indicate that the regulation of bone genes via lncRNA may provide novel opportunities for bone disease therapy.

## Epigenetic therapy for BMSC dysfunction in age-related bone diseases

**Fig. 1:**
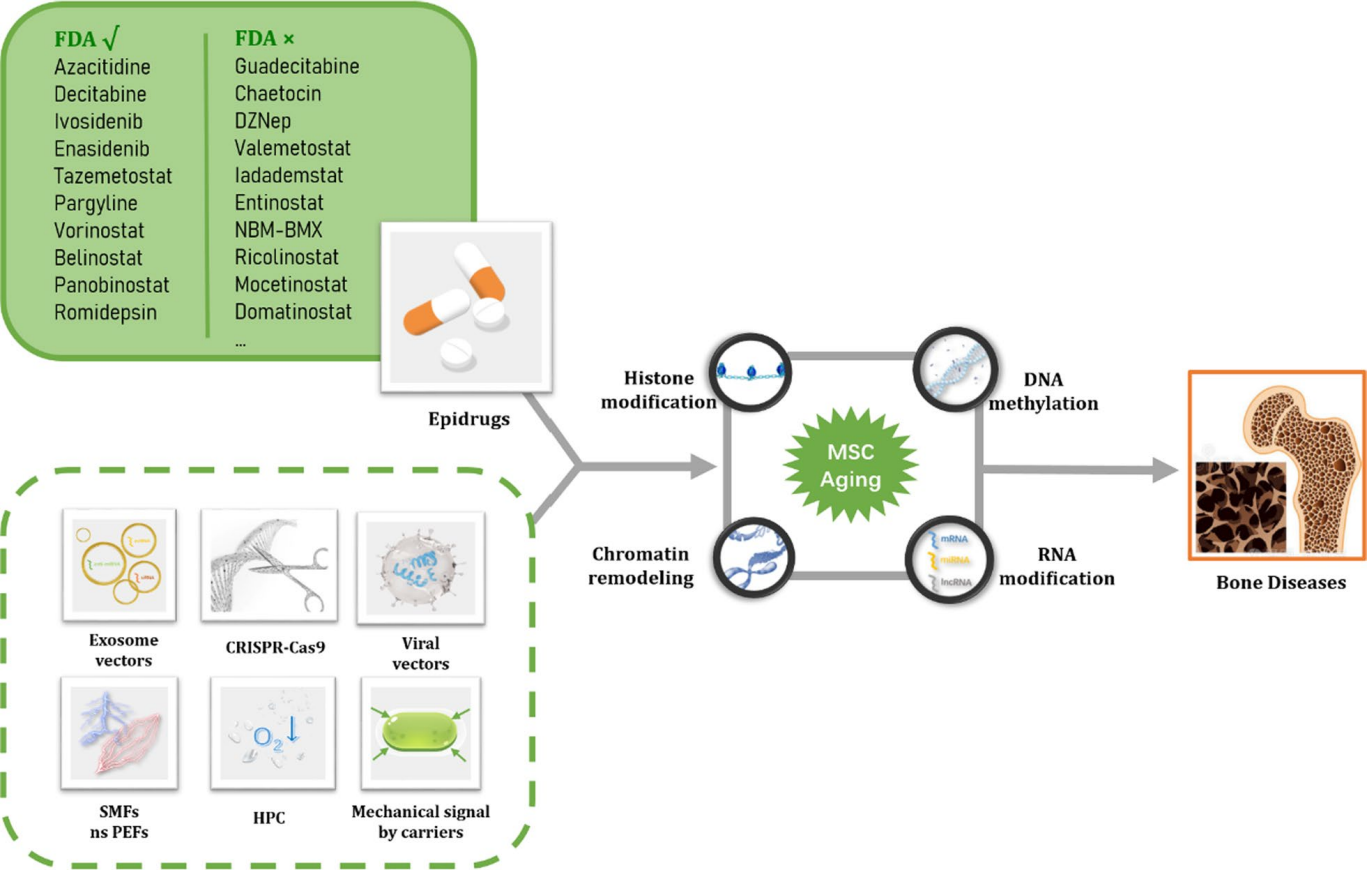
Epigenetic therapies for BMSC aging in age-related bone diseases. Epigenetic regulation for BMSC aging entails DNA methylation, histone modification, chromatin remodeling, and RNA modification, and therapeutic interventions for age-related bone diseases are various. Some epi-drugs have been approved by the Food and Drug Administration (FDA) and used in clinics extensively, and others are in the experimental stage. There are also potential therapeutic interventions using epi-drugs to enhance therapeutic effects, which still have a long way to go before being clinically utilized. Such therapeutic interventions include genetic editing therapy involving CRISPR-Cas9, viral vectors, and exosome vectors, and adjuvant therapy including static magnetic fields (SMFs), nanosecond pulsed electric fields (nsPEFs), hypoxic preconditioning (HPC), and mechanical signal by carriers.

In the last decade, several strategies of epigenetic therapy have been developed. Basically, these strategies include epigenetic drugs, gene editing techniques, and adjuvant therapy including mechanical signal, hypoxia therapy, and magnetic and electric therapy (Fig. 1).

### Epigenetic drugs controlling the activity of epigenetic enzymes

Epigenetic modifications of BMSCs can be controlled by regulating the levels and activity of epigenetic enzymes, which leads to chromatin structure alterations and influences the expression of aging and anti-aging genes [89]. An attractive strategy is to use chemical modifiers of epigenetic enzymes, called epi-drugs, to regulate the epigenetics of BMSCs [95]. Against the epigenetic therapeutic targets described above, several chemical modifiers have been investigated. For example, the inhibitor of DNMT, 5-Aza-2'-deoxycytidine (also known as decitabine), is effective in delaying BMSC aging [35]. The HDAC inhibitor, TSA, can promote osteogenic differentiation of rat adipose-derived MSCs [49]. The EZH2 inhibitors, 3-deazaneplanocin A (DZNep) and GSK126, can induce osteogenesis while suppress excessive bone marrow fat formation, leading to alleviation of OP in mice [42, 43]. Other chemical modifiers possessing the potential to combat MSC aging and promote osteogenesis are listed in Table 3.

**Table 3:** Epigenetic drugs

Enzyme	Inhibitor drugs	FDA	Therapeutic effect	Osteogenesis mechanism	References
DNMT	Azacitidine	√	Chronic myelomonocytic leukemia	–	
			Myelodysplastic syndrome	–	
	Decitabine	√	Chronic myelomonocytic leukemia	–	
			Myelodysplastic syndrome	–	
			Promoting osteogenesis	SOD2 → ROS↓	[34–36]
	RG108	–	Improving BMSC migration	TERT, bFGF,	[99]
			Delaying BMSC senescence	VEGF, ANG	
	Guadecitabine	–	cancer therapy possibility	–	
IDH1	Ivosidenib	√	Oncometabolite therapy	–	
IDH2	Enasidenib	√	Oncometabolite therapy	–	
SUV39h1/2	Chaetocin	–	Promoting osteogenesis	Wnt/β–catenin	[41]
EZH2	Tazemetostat	√	Follicular lymphoma	–	
	DZNep	–	Promoting osteogenesis	Wnt/β–catenin	[42]
	GSK126	–	Promoting osteogenesis	BMP2	[43, 44]
	Valemetostat	–	Cancer therapy possibility	–	
	CPI-1205	–	Cancer therapy possibility	–	
	CPI-0209	–	Cancer therapy possibility	–	
KDM5A	JIB-04	–	Promoting osteogenesis	BMP2	[46]
LSD1	Pargyline	√	Decrease blood pressure	–	
			Promoting Osteogenesis	H3K4	[47]
	Iadademstat	–	Acute myelomonocytic leukemia	–	
			Myelodysplastic syndrome	–	
	CC-90011	–	Cancer therapy possibility	–	
	INCB059872	–	Cancer therapy possibility	–	
HDAC I/II	Vorinostat	√	Cutaneous T cell lymphomas	–	
			Promoting osteogenesis	Runx2, BMP	[50]
	Belinostat	√	Peripheral T cell lymphomas	–	
	Panobinostat	√	Multiple myeloma	–	
	Romidepsin	√	Cutaneous T cell lymphomas	–	
			Peripheral T cell lymphomas	–	
	Chidamide	*	T cell lymphomas	–	
	TSA	–	Promoting osteogenesis	Runx2, BMP	[49]
			Delaying BMSC senescence	NF–κB (p65)	
	Entinostat	–	Cancer therapy possibility	–	
	NBM-BMX	–	Cancer therapy possibility	–	
	Ricolinostat	–	Cancer therapy possibility	–	
	Mocetinostat	–	–	–	
	Domatinostat	–	–	–	
	Pracinostat	–	–	–	
	OKI-179	–	–	–	
	Givinostat	–	–	–	
	Abexinostat	–	–	–	
	Resminostat	–	–	–	
	Fimepinostat	–	–	–	
	Citarinostat	–	–	–	
	KA2507	–	–	–	
HDACIII	Resveratrol		Cartilage protection	SIRT1 → HIF–2α↓	[100–102]
BRD4	Molibresib Birabresib	– –	NUT midline carcinoma NUT midline carcinoma	– –	

^*^Chidamide has received regulatory approval in China

Epigenetic drugs are generally epigenetic enzyme inhibitors, affecting DNA and histone modifications and leading to chromatin structure changes. Most epi-drugs listed above are in preclinical and clinical development, and two DNMT inhibitors, four HDAC inhibitors, two IDH inhibitors, one EZH2 inhibitor, and one LSD1 inhibitor have been approved by the FDA available for standard-of-care treatment for cancer. However, these epi-drugs are mainly used to treat cancers, and only a small number of epi-drugs have been studied in terms of BMSC anti-aging and osteogenesis. Further research is needed

SOD2, superoxide dismutase 2; ROS, reactive oxygen species; TERT, telomerase reverse transcriptase; FGF, fibroblast growth factor; VEGF, vascular endothelial growth factor; ANG, angiogenin; DZNep, 3-deazaneplanocin A; BMP, bone morphogenetic protein; TSA, trichostatin A; SIRT1, silent information regulator 2 type 1; HIF, hypoxia-inducible factor; NUT, nuclear protein of the testis

However, the application of these drugs in age-related bone diseases has not been evaluated in clinical studies yet. By searching “epigenetic therapy AND (osteoporosis OR osteoarthritis OR aging OR anti-aging)” on the database of Clinical Trials, the results showed only two ongoing clinical trials. One is a comprehensive treatment involving exercise, nutrition, bisphosphonates, statins, calcitonin, and vitamin D, with an examination of epigenetic changes in the aging condition of the nervous system. The other is a small ongoing clinical trial about quercetin, a natural flavonoid, and dasatinib, a poly-tyrosine kinase inhibitor, to adjust the epigenetic age frame for anti-aging therapy. Up to now, no clinical trial about epigenetic therapy on bone diseases has been carried out. Therefore, clinical research in this area is in urgent demand.

At present, some epigenetic drugs have been approved by the Food and Drug Administration (FDA) for clinical treatment, [96] but they are mainly used to treat cancer. These drugs include two DNMT inhibitors, four HDAC inhibitors, two IDH inhibitors, one EZH2 inhibitor, and one LSD1 inhibitor. DNMT inhibitors azacitidine and decitabine were developed and then approved long before the discovery of their methylation mechanism. They are both antimetabolites, which inhibit DNMT activity and induce hypomethylation when incorporated with DNA. They were approved for the treatment of myelodysplastic syndrome and chronic myelomonocytic leukemia [97, 98]. The four HDAC inhibitors approved by the FDA are vorinostat, romidepsin, belinostat, and panobinostat. Another HDAC inhibitor chidamide has also been approved in China [96]. HDAC inhibitors directly bind to HDAC to prevent lysine deacetylation. By neutralizing positive charges on lysines, acetylation reduces the electrostatic attraction of lysines to negatively charged DNA, thereby loosening chromatin and promoting gene transcription. HDAC inhibitors are mainly used for the treatment of T cell lymphomas. In addition, there are also some other epi-drugs developed and approved by FDA in recent years, such as ivosidenib, enasidenib, and tazemetostat, which are used clinically to treat cancer.

The research on anticancer epigenetic drugs has been relatively mature, and more epigenetic-related enzyme inhibitors are under development. However, whether these epigenetic drugs used for cancer treatment can be used for the treatment of age-related bone diseases requires further research.

### Genetic editing technology regulating epigenetic-related genes

Gene editing therapy is to regulate specific gene expression in specific cells to treat pathological conditions by introducing exogenous nucleic acids, such as DNA, mRNA, small interfering RNA (siRNA), micro-RNA (miRNA), or antisense oligonucleotides into target cells. Recently, the techniques of clustered regularly interspaced short palindromic repeats/CRISPR-associated protein 9 (CRISPR-Cas9) and RNA interference (RNAi) have been tested in the treatment of BMSC aging. CRISPR-Cas9 is an adaptive immune defense system formed by bacteria in evolution to combat invasive viruses and exogenous DNA. CRISPR-Cas9 has been proved as a powerful technique to splice targeted DNA [103]. Screening with CRISPR-Cas9 technology can be used to discover age-related genes (such as *KAT7*) systematically. Genome-editing strategies based on CRISPR/Cas9 are also a promising approach to treating age-related diseases. For example, KAT7, a histone acetyltransferase acetylates histone H4, is involved in the activation of certain transcription-related genes. It helps to unfold chromatin so that DNA can be accessed and copied or transcribed, which is necessary for a functional replication origin. Intravenous administration of lentiviral vector encoding Cas9/sg-*Kat7* slows down liver aging and prolongs the life span of elderly mice [45]. The anti-aging effect of KAT7 could also be studied in the field of age-related bone diseases.

As one of the main defense mechanisms against pathogens in multiple organisms, RNA interference (RNAi) is a process to silence target mRNA. The tool of RNAi is small double-stranded RNA (about 21nt length), including endogenous miRNAs or exogenous siRNA [104]. An example of miRNA treatment is that down-regulation of miR-29a-3p/miR-30c-5p in human BMSCs resulted in up-regulation of DNMT3A, hypermethylation of SOD2 upstream CpG islands, and down-regulation of SOD2, which accelerated senescence [35]. siRNA treatment can also silence specific genes related to aging, which may attain therapeutic effects [105]. Knockout of histone demethylase (Jarid1a) by siRNA can significantly improve the expression of bone-specific mRNA and protein and enhance osteogenic differentiation of BMSCs in vitro [48]. To improve the selectivity of epigenetic regulation, artificial transcription factors have been engineered by the fusion of a DNA-binding domain to one or more effector domains to enable accurate gene activation and repression. ATFs including linked zinc finger modules, transcription activator-like effectors, and RNA-guided systems based on CRISPR/Cas9 are powerful tools to facilitate selective epigenetic regulation [106].

The above carrier-free gene therapies have the advantages of lower manufacturing cost and less immunogenicity [107]. However, their shortcomings are limited targeting capability, instability in vivo due to nuclease, and lower treatment efficiency owing to the difficulty of entering cells and escaping from lysosomes. To enhance the selectivity of epigenetic regulation and require precise in vivo delivery, gene therapy requires the help of vectors. Nevertheless, the success of epigenetic engineering strategies is highly dependent on the collateral development of safe, immune inert, and targeted delivery systems to enable CRISPR-Cas9, RNAi, and other exogenous nucleic acids expression in specific cell types, such as BMSCs. For example, lentivirus is a single-stranded RNA (ssRNA) virus that belongs to a family of retroviruses, which can transfer and integrate targeting genes into recipient cells [108–110]. Transfection of anti-miR-31 by lentiviral vectors induces bone formation in BMSCs, demonstrating its therapeutic potential [73]. Exosomes carry cargos such as miRNAs, DNA, mRNAs, and proteins from source cells to protect them from being degraded by ribonuclease, ensuring their epigenetic regulatory effects. By transporting via exosomes, miRNAs related to anti-aging and osteogenesis, such as miR-148 [67] and miR-328-3p [68], may achieve increased therapeutic effects. Although evidence about the application of exosomes in age-related bone diseases is insufficient, it is worth further investigation. Also, nanotechnology represents a valid alternative due to the flexibility of the scaffolds. Modified nanoparticles could allow large cargos to be accommodated, render the nanoparticles immunologically inert, reduce their immune clearance, stimulate cell internalization or endocytosis, and promote endosomal escape [106]. Recently, structured DNA assemblies fabricated using the principle of scaffolded DNA origami have been applied as a new nonviral delivery vector. The vector has the properties of controllable immunostimulatory, virus-like spatial presentation of ligands and immunogens for cell-specific targeting, intracellular trafficking, and low manufacturing cost, providing a novel tool for delivery of nucleic acids [111].

### Adjuvant treatment

#### Mechanical signal

Stem cell engineering currently uses mechanical signals in the microenvironment to regulate stem cell behaviors. Cells perceive and transmit mechanical signals from the extracellular matrix to the cytoskeleton and nuclear lamina, which is considered a key mechanical sensor that can directly affect chromatin structure, gene expression, and epigenetic modification. Experiments have shown that through extracellular carriers, such as photosensitive hydrogels [112] and dendrimer-immobilized surface [113], mechanical signals can be transmitted to BMSCs. The enhanced hardness of the extracellular matrix leads to bone formation of BMSCs. Mechanical signals increase nuclear tension and then promote histone acetylation by inactivating histone deacetylases (HDAC). And the BMSCs from bones of OA patients have shown nuclear mechanical defects with similar mechanisms involving defective nuclear mechano-sensing and HDAC up-regulation. This indicates that nuclear mechanical sensor controls BMSC osteogenesis by mediating HDAC epigenetic remodeling, providing potential therapeutic targets for age-related disease [112].

#### Hypoxia therapy

Hypoxic preconditioning (HPC) is beneficial to cell proliferation, differentiation, and gene expression. It has been clear that compared with air oxygen (21% O_2_), HIF and SOX2 are up-regulated under hypoxic conditions (2% O_2_), which may decelerate the aging of BMSCs, but its epigenetic mechanisms are still unknown. Nowadays, BMSC epigenetic changes after hypoxic preconditioning are shown in a study; 5mC and 5hmC decreased as methylation increased on *Dnmt3B* and *Tet1* promoters, reducing expression of DNMT3B and TET1 [114]. It shows that HPC for BMSC anti-aging therapy may entail a potential mechanism in epigenetic regulation, but the specific connection still needs further research.

#### Magnetic and electric therapy

Magnetic therapy based on static magnetic fields (SMFs) may be an optional adjuvant treatment for patients with OP. SMF (0.2–0.6 T) promotes BMSC osteogenesis and inhibits adipogenic differentiation in an intensity-dependent manner. RNA-seq analysis of the entire genome in BMSCs has shown that SMF (0.6 T) could reduce PPARγ expression and increase RUNX2 transcription [115]. Moreover, recent research also reported a novel and effective strategy, namely nanosecond pulsed electric fields (nsPEFs) stimulation. Via down-regulation of DNMT1, nsPEFs lead to demethylation of the promoters of genes OCT4 and NANOG, which results in increased expression of these two genes and enhanced BMSC differentiation toward various directions, including bone, cartilage, and fat [116].

## Epigenetic therapy for age-related skeletal diseases

### Osteoporosis

OP is an age-related metabolic disease characterized by low bone density, deterioration of the bone structure, and the occurrence of fragility fractures. It is characterized by bone loss and fat accumulation in bone marrow, which inhibits the maturation of osteoblasts. Fractures caused by OP are becoming increasingly common among women over 55 years old and men over 65, leading to higher healthcare costs and mortality [117]. The current treatment is mainly anti-absorption drugs (e.g., bisphosphonates and denosumab) and anabolic drugs (e.g., teriparatide and abalopatide) [118]. But these drugs are associated with limited long-term efficacy and relatively severe side effects [119]. Meanwhile, epigenetic therapy for BMSCs of OP patients provides a potential alternative.

Epigenetic regulation of methylation is important for regulation of BMSC differentiation. Recent studies have shown that chaetocin, an inhibitor of methyltransferase SUV39h1/2, affected BMSC differentiation, leading to increased osteogenesis and decreased adipogenesis. During osteogenic induction, chaetocin induced the expression of osteogenic markers (Runx2 and OPN) in BMSCs and improved Wnt/β-catenin signaling pathway and its downstream targets [41]. In addition, the combined application of bone morphogenetic protein 2 (BMP2) and methyltransferase EZH2 inhibitors optimized the therapeutic effect on osteoporosis. Therefore, a new strategy is proposed that combination with EZH2 inhibitors could minimize the dosage of BMP2, which reduces the adverse events and high cost when BMP2 is used alone. The experiment showed that co-administration of BMP2 and EZH2 inhibitor GSK126 enhanced the differentiation ability of mouse osteoblasts [43, 44]. Other osteogenic potential Ezh2 inhibitors (iEzh2) have also been studied, increasing the potential choices of bone-promoting drugs. For example, M. L. Galvan et al. examined a group of iEzh2s and suggested that all eight inhibitors (EPZ-6438, GSK126, PF-06726304, UNC1999, UNC, GSK503, EI1, and CPI-169) accelerated osteoblast differentiation to different degrees, at concentrations far below cytotoxicity [120]. Loss of KDM4B in BMSCs increased marrow adiposity via increasing H3K9me3, reducing bone formation, and exacerbating skeletal aging and OP, which may serve as a new target for BMSCs to prevent and treat bone aging [39].

Epigenetic regulation of acetylation is of equal significance. Histone 3 acetylation is involved in maintaining bone homeostasis. By treating osteoblasts with HDAC inhibitor, TSA, the expression of acetyl-histone 3 was up-regulated, and the osteogenic ability of osteoblasts differentiated from BMSCs was restored [121]. Glucocorticoid can inhibit H3K9ac at Runx2 promoter, causing bone loss and bone marrow adipogenesis, and accelerating OP. Bromodomain protein BRD4 is a chromatin reader that binds acetyl-histone and regulates the homeostasis of stem cells. In vivo, BRD4 inhibitor JQ-1 treatment reduced the inhibitory effect of methylprednisolone on bone trabecular mineralization and osteogenic differentiation, reversing bone marrow fat and adipocyte formation [122].

Individuals with a higher rate of body fat (independent of body weight) have an increased risk of OP, osteopenia, and non-spinal fractures [123]. Obesity can damage the repair ability of BMSCs and cause dysfunction by disturbing their normal transcription, protein synthesis, and paracrine function. Using epigenetic regulator VIT-C, some of these changes may be reversible. Experiments showed that the whole-genome epigenetics of BMSCs in obese pigs changed. After co-culture with VIT-C, 5hmC was enhanced and overall levels of H3K9me3 and H3K27me3 were decreased [124]. In addition, histone demethylase KDM4D is a key regulator of adipogenesis, and the depletion of KDM4D leads to damage of BMSC adipogenic differentiation, which may also become a potential therapeutic target [125].

### Osteoarthritis

Osteoarthritis (OA) is the most common joint disease characterized by progressive degradation of articular cartilage, leading to joint dysfunction, pain, and disability [126]. Aging is one of the main factors contributing to OA pathogenesis via releasing senescence-associated secretory factors by increasing senescent cells in joints [23]. Current treatments for OA include medication and surgery. Traditional medications include steroidal or nonsteroidal anti-inflammatory drugs, which can relieve pain and inflammation [127]. Hyaluronic acid injection in the joint cavity can protect the articular cartilage tissue and improve lubrication. There are also emerging drug treatments, disease-modifying OA drugs, which regulate the degenerative changes of OA cartilage by targeting inflammatory cytokines, matrix-degrading enzymes, Wnt pathways, and targeting pain caused by OA [128]. Surgical treatment includes arthroscopic joint debridement, arthrodesis, joint replacement, etc., to eliminate pain, correct deformity, and improve joint function. There are also some physical assistance therapies, such as oxygen-ozone therapy, hyperthermia, hydrotherapy, and acupuncture, which can improve joint function to a certain extent. However, the curative effect is unsatisfactory in a certain percentage of patients. Precise and personalized therapy remains the ultimate yet unaccomplished goal [129].

Recent studies have proved that epigenetic is vital to cartilage health and homeostasis. In the process of cartilage formation, methylation changes are distinctive in BMSCs. Especially in the enhancers marked by characteristic histone modifications (H3K4me1 and H3K27ac), abundant DNA demethylation occurs, while the CpGs of these enhancers are highly methylated in other tissues [130, 131]. Researchers have also discovered new clues between epigenetic pathophysiology and OA. Expression of DNMT3b decreases in chondrocytes of OA mice and human OA patients, leading to thorough demethylation. Targeted deletion of DNMT3b in chondrocytes leads to OA. Moreover, after DNMT3b function was obtained in mouse articular chondrocytes in vitro and in vivo, chondroprotection was observed, indicating that DNMT3b contributes to the homeostasis of articular cartilage. Therefore, the cellular pathway regulated by DNMT3b may provide a novel target for the treatment of OA [33].

Histone acetylation may also be a promising target to promote cartilage repair in OA treatment. HDACs promote normal cartilage development and homeostasis. In HDAC3-deficient chondrocytes, obstacles in extracellular matrix production, bone development, and maturation of chondrocytes were observed [132]. HDAC4 and HDAC5 can suppress chondrocyte hypertrophy by inhibiting Runx2 signaling cascade [133]. HDAC7 inhibits the proliferation of chondrocytes and β-catenin activity, and reducing HDAC7 levels in chondrocytes in the early stage may promote the expansion and regeneration of cartilage tissue [134]. TSA and vorinostat inhibit the activity of HDACs classic I and II, protecting cartilage by reducing MMP [135–137]. TSA also increases the expression of Nrf2, a transcription factor that regulates the expression of phase II antioxidant enzymes, to protect against oxidative stress and tissue damage in mouse joint tissues [137]. In addition to the classic HDACs (including class I, II, and IV) mentioned above, HDAC class III proteins, called sirtuins, are also involved in the protective process in OA. The destruction of cartilage can be inhibited by increasing the activity of SIRT1 and SIRT6 in OA mice [138–140]. Intra-articular injection of resveratrol, an activator of SIRT1, can alleviate the destruction of OA cartilage by inhibiting the expression of HIF-2α and catabolic factors [100]. Moreover, resveratrol has also been studied to inhibit the other three types of HDAC (class I, II, and IV) [101]. But most of the studies are focused on chondrocytes. The effect of HDACs on BMSCs might be prosperous and still needs further research.

In addition to histone regulation, lncRNA CIR also participates in OA. Knocking down lncRNA CIR or over-expressing ATOH8 promotes BMSC cartilage formation through EZH2-mediated epigenetic modifications [82]. Studies have shown that miRNA is differently expressed in the cartilage of OA patients compared with the normal cartilage. Some miRNAs (e.g., miR-9, miR-18a, miR-22, miR-60) are up-regulated, [141–144], while others (e.g., miR-27, miR-140, miR-146, 149-5p, miR-199a, miR-miR-602, miR-608) are down-regulated [145–150]. The changes influence various target genes and numerous physiological mechanisms, including lipid metabolism, matrix degradation, inflammation, and chondrocytes differentiation. Although several studies suggest miRNAs are potential treatment targets of OA, the application of miRNA-based treatment for OA is relatively rare. A study has shown that a JAK inhibitor, tofacitinib, has therapeutic potential in OA by up-regulating the levels of miR-149-5p [146].

### Inflammatory bone loss in periodontitis

Epigenetic regulation of BMSCs can also be used to treat periodontitis by promoting osteogenesis. Periodontitis is a common chronic infection of periodontal tissue that ends up with the loss of teeth in the elderly. Dysfunction of stem cells and impaired immune regulation may lead to poor repair of periodontal tissue. A recent study from Q. Li al. indicates that epigenetic modifiers have a desirable effect on regulating the fate of BMSCs in the inflammatory microenvironment, which is beneficial for the treatment of periodontitis. TSA rescues the defect of osteogenesis of BMSCs isolated from inflammatory gingival tissues by inhibiting the bind of nuclear factor-κB (p65) to DNA in BMSCs. Treatment of TSA significantly increased alveolar bone mass and inhibited inflammatory infiltration, indicating that TSA is a potential therapeutic option for periodontal tissue repair [151].

## Discussion and prospect

### Network complexity and precise epigenetic therapy

A single epigenetic enzyme can regulate multiple genes and play different roles in different situations, exerting auxiliary or synergistic effects. The coordination and antagonization of multiple epigenetic enzymes construct a complex epigenetic regulatory network. Therefore, we need a deeper and more precise understanding of the epigenetic network of MSCs during aging. Epigenetic regulation is complex and accurate, but the effect of current epigenetic drugs is general rather than precise. They inhibit a whole class of epigenetic enzymes and affect almost the entire genome's epigenetic modification. Some modifications may have a therapeutic effect, some may be ineffective changes, and some may even cause harmful consequences. As the current epigenetic drugs still lack cell specificity, how to improve the precise therapy for BMSC aging remains a challenge. So far, plenty of efforts have been made to enhance the selectivity of epigenetic regulation and in vivo delivery. For example, targeted delivery systems can carry epigenetic drugs to the desired cells and tissues. Gene editing techniques based on lineage-specific transcriptional factors or markers are potential to change the aberrant epigenetic modifications in aged BMSCs.

### Potential side effects of epigenetic drug therapy

At present, clinical trials of epigenetic therapy for anti-aging are in shortage. But epigenetic drugs for anticancer have been widely tested in trials and applied in the clinic. Cancer is caused by the accumulation of many tiny genetic mutations, and similarly, gene damages gradually appear in the process of aging. There are many similarities in epigenetic regulation between aging and cancer. In several cancers, the decline of heterochromatin DNA methylation and inhibitory histone modification leads to chromatin instability, while increased DNA methylation in growth-related and tumor suppressor gene promoters leads to abnormal cell function. Therefore, based on a similar mechanism, it is reasonable to apply anticancer epigenetic drugs to age-related diseases.

However, several problems remain to be solved before anticancer epigenetic drugs can be clinically applied to age-related diseases. Considering the differences of severity between cancer and age-related bone diseases, patients with age-related bone diseases are less likely to accept the side effects of drugs. Besides, epigenetic therapy only changes abnormal epigenetic modifications but cannot reverse gene damages. As a result, epigenetic therapy is more efficient in the early stage of diseases. However, considering that prevention and early treatment are critical in age-related bone diseases, epigenetic therapy generally indicates a promising prospect.

### Combination of epigenetic therapy with other anti-osteoporosis treatment

In the early stage, senescence entails the accumulation of abnormal genetic modifications. Simultaneously, aging leads to genetic damage in stem cells. Epigenetic regulation can reverse the accumulation of genetic modification abnormalities to a certain extent, but it cannot reverse genetic defects. This grants epigenetic modulators a cutting-edge in early disease prevention, but during disease progression, epigenetic modulators alone may be inadequate. Enlightened by current tumor treatment, epigenetic modulators are mostly used in combination with other anti-tumor therapy. The combined use of multiple therapies may play a complementary and enhanced role. The combined application of epigenetic drugs is also promising in the future anti-aging treatments for skeleton. But the interactions between epigenetic drugs and existing anti-aging drugs still need further study to ensure safety. In addition, some non-drug therapies are expected to exert synergistic effects. Gene editing technology is promising, though many obstacles remain to be combated before clinical application. More adjuvant treatment methods such as mechanical action, hypoxia, and electromagnetic stimulation are also being explored. We expect that the exploration of various combination therapies can bring about new treatment directions in the future.

## Conclusions

Alteration in epigenetic modification is an essential factor of BMSC dysfunction during aging. The transferability and reversibility of epigenetic regulation provide the possibility to combat BMSC aging. Many epigenetic enzymes and regulators have been demonstrated as potential therapeutic targets for BMSC aging. Emerging evidence demonstrates that epigenetic therapy based on aberrant epigenetic modifications could alleviate the senescence and dysfunction of stem cells, leading to the alleviation of age-related bone diseases. Along with the rapid development of epigenetic therapeutic strategies, epigenetic therapy targeting BMSCs is promising in the clinical application of age-related bone diseases.

## Data Availability

All data generated or analyzed in the manuscript are included in this article.

## Abbreviations

5mC: 5-Methylcytosine
5hmC: 5-Hydroxymethylcytosine
ALKBH: AlkB homolog
ANG: Angiogenin
ATOH8: Atonal bHLH transcription factor 8
BMSC: Bone marrow mesenchymal stem cell
BMP: Bone morphogenetic protein
BRM: Brahma
BRD4: Bromodomain-containing protein 4
CBX4: Chromobox4
DNMT: DNA methyltransferase
DZNep: 3-Deazaneplanocin A
EZH: Enhancer of zeste homolog
FGF: Fibroblast growth factor
G9a: A lysine-specific histone methyltransferase
GCN, also known as Eif2ak4: Eukaryotic translation initiation factor 2 alpha kinase 4
FDA: Food and Drug Administration
FTO: FTO alpha-ketoglutarate-dependent dioxygenase
HDAC: Histone deacetylase
HIF: Hypoxia-inducible factor
HPC: Hypoxic preconditioning
hASC: Human adipose-derived stem cell
hUC-MSC: Human umbilical cord mesenchymal stem cell
iPSC: Induced pluripotent stem cell
LSD, also known as KDM: Lysine-specific demethylase
lncRNA: Long noncoding RNA
METTL: Methyltransferase-like
MSC: Mesenchymal stem cell
mRNA: Message RNA
miRNA: Micro-RNA
N6mA: N6-methyladenine
NaBu: Sodium butyrate
NANOG: Nanog homeobox
Nrf2: Nuclear factor (erythroid-derived 2)-like 2
nsPEF: Nanosecond pulsed electric field
OA: Osteoarthritis
OCT4: Organic cation/carnitine transporter 4
OP: Osteoporosis
ROS: Reactive oxygen species
PTNE: Phosphatase and tensin homolog
RNAi: RNA interference
SIN3A: SIN3 transcription regulator family member A
SIR: Silent information regulator
SMF: Static magnetic field
SOD2: Superoxide dismutase 2
SOX2: SRY-box transcription factor 2
SUV39H: A lysine-specific histone methyltransferase
siRNA: Small interfering RNA
ssRNA: Single-stranded RNA
TERT: Telomerase reverse transcriptase
TET: Ten-eleven translocation family protein
TSA: Trichostatin A
VEGF: Vascular endothelial growth factor
VPA: Valproate
VIT-C: Vitamin C
WTAP: Wilm's tumor-associated protein
ZKSCAN3: Zinc finger with KRAB and SCAN domains 3
